# FRAILTY AS A PREDICTOR OF MORTALITY: A COMPARATIVE COHORT STUDY IN COSTA RICA AND THE UNITED STATES

**DOI:** 10.1093/geroni/igad104.3360

**Published:** 2023-12-21

**Authors:** Carolina Santamaría-Ulloa, Amanda Lehning, Mónica Cortés-Ortiz, Ericka Méndez-Chacón

**Affiliations:** Universidad de Costa Rica, San Jose, San Jose, Costa Rica; University of Maryland Baltimore, Baltimore, Maryland, United States; University of Maryland Baltimore, Baltimore, Maryland, United States; Universidad de Costa Rica, San Jose, San Jose, Costa Rica

## Abstract

Geriatric frailty results from aging-related declines in multiple systems and leads to increased vulnerability for loss of mobility, falls, hospitalization, and mortality. The aim of this study is to compare the association between frailty and mortality in older adults from Costa Rica and the United States. This prospective cohort study uses secondary nationally representative data of community-dwelling older adults from the Costa Rican Longevity and Healthy Aging Study (CRELES, n = 2124) and the National Health & Aging Trends Study (NHATS, n = 6,680). Frailty status was assessed using Physical Frailty Phenotype, which includes shrinking, exhaustion, weakness, slowness, and low physical activity level. We estimated Cox proportional hazard models to examine the association between frailty and all-cause mortality at 8 years from the date at baseline. Survey weights were used. Sociodemographic characteristics and health behaviors were included as covariates. The death hazard for frail compared to non-frail older adults was three-fold in Costa Rica (HR = 3.14, p< 0.001) and four-fold in the white US (HR = 4.02, p< 0.001). Older age, being male, and smoking were death risk factors in both countries. High education was a protective factor in the US, whereas being married or in union was a protective factor in Costa Rica. Results indicate that frailty can have a differential impact on mortality depending on the context of different countries. The identification of behavioral, social, and policy determinants of frailty may serve as an essential component of population interventions that prevent the progression of frailty and its consequences.

